# One size does not fit all–qualitative process evaluation of the Healthy School Start parental support programme to prevent overweight and obesity among children in disadvantaged areas in Sweden

**DOI:** 10.1186/s12889-016-2701-1

**Published:** 2016-01-14

**Authors:** Åsa Norman, Gisela Nyberg, Liselotte Schäfer Elinder, Anita Berlin

**Affiliations:** 1 Department of Public Health Sciences, Karolinska Institutet, 171 77 Stockholm, Sweden; 2 Department of Neurobiology, Care Sciences and Society, Karolinska Institutet, Box 23100, 141 83 Huddinge, Sweden; 3 Centre for Epidemiology and Community Medicine, Stockholm County Council, Box 1497, 171 29 Solna, Sweden

**Keywords:** A Healthy School Start, Implementation, School, Intervention, Motivational interviewing, Diet, Physical activity, Health promotion, CFIR, Content analysis

## Abstract

**Background:**

Parental support interventions have shown some effectiveness in improving children’s dietary and physical activity habits and preventing overweight and obesity. To date, there is limited research on barriers and facilitators of school-based parental support interventions targeting overweight and obesity. This study aimed to describe barriers and facilitators influencing implementation of the Healthy School Start (HSS) intervention in disadvantaged areas in Stockholm, Sweden, from the perspective of parents and teachers.

**Methods:**

Focus groups and individual interviews with teachers (*n* = 10) and focus groups with parents (*n* = 14) in the intervention group of the HSS were undertaken, guided by the Consolidated Framework for Implementation Research (CFIR). Transcriptions were analysed using qualitative content analysis in two steps: deductive sorting in two domains of the CFIR (intervention characteristics and process), and subsequent inductive analysis.

**Results:**

The overarching theme “tailoring the intervention to increase participant engagement” was found. Among teachers, barriers and facilitators were related to how the intervention was introduced, perceptions of the usefulness of the classroom material, preparation ahead of the start of the intervention, cooperation between home and school and children’s and parents’ active engagement in the intervention activities.

For parents, barriers and facilitators were related to the perceived relevance of the intervention, usefulness of the material, experiences of the Motivational Interviewing (MI) sessions, the family member targeted by the intervention, cooperation between home and school and parents’ ability to act as good role models.

**Conclusion:**

It seems important to tailor the intervention to the abilities of the target group in order to increase participant engagement. Including activities that focus on parents as role models and cooperation between parents seems important to bring about changes in the home environment. It also appears important to include activities that target cooperation between home and school.

## Background

Overweight and obesity affects groups with low socio-economic status (SES) in Sweden to a higher extent, both adults [1] and children [2]. In Sweden, the prevalence of overweight is 13 % and obesity 2.6 % in 7 to 9-year-old children [2]. The prevalence of obesity in 10-year-old children in deprived areas has been shown to be three times higher compared to affluent areas [3]. Parental support interventions have been partially effective in improving dietary and physical activity habits and in preventing overweight and obesity [4–6].

To date, there is limited research regarding barriers and facilitators to implementation of parental support interventions targeting overweight and obesity in general and in particular for such interventions taking place in the school context.

Previous studies regarding school-based obesity prevention interventions have found that complexity and lack of clarity of the intervention, time constraints and lack of deliverers’ training constitute barriers to implementation, whereas a detailed programme manual, external support and technical assistance may act as facilitators [7–9].

In a qualitative review on barriers and facilitators of parents’ participation in general parenting interventions, barriers for the parents included group dynamics, parents’ time and resources, stigma related to gender, social status, accessibility of venue, didactic delivery of intervention, and participants’ lifestyle. Parents found that learning new skills from a trustworthy deliverer, meeting others and exchanging ideas, interventions tailored to the individual, as well as suitable timing and location facilitated participation [10]. Deliverers perceived participants’ lifestyles, deliverers’ training and skills and cultural context as barriers, whereas interventions tailored to the individual and proper training of the deliverers facilitated parental participation [10].

The Healthy School Start (HSS) is a school-based parental support programme promoting healthy dietary and physical activity habits to prevent child overweight and obesity in disadvantaged areas [11]. The programme has to date been evaluated in two cluster randomised wait-list controlled trials in areas with medium to low SES [12]. The present study describes the process evaluation of the second trial carried out in areas with low SES in Stockholm County. Barriers and facilitators to implementation of the programme have been investigated by using the Consolidated Framework for Implementation Research (CFIR) [13]. CFIR comprises five domains: intervention characteristics, outer setting, inner setting, characteristics of individuals, and process. This study focuses on intervention characteristics and process, as these two domains appeared to be thematically related to each other during the data analysis. Within the domain intervention characteristics, important constructs comprise adaptability to local needs, whether the intervention is developed by the users or by an external organisation, perceived relative advantage, complexity, trialability, evidence strength, cost and design. Regarding the process domain, constructs crucial for successful implementation include how the planning, execution and evaluation are undertaken as well as how the deliverers are engaged in the intervention.

The process evaluation of the first HSS trial, conducted in an area with low to medium SES in Stockholm during 2010–2011, showed that clear communication between teachers and parents together with well-defined roles were crucial to implementation [14]. The aim of the present study was to describe barriers and facilitators, related to intervention characteristics and process, that teachers and parents in a disadvantaged setting perceived as influencing the implementation of the HSS intervention. This process evaluation fills an important gap regarding factors of importance for implementation of obesity prevention programmes in disadvantaged areas including both the school and home.

## Methods

### Study design

A qualitative descriptive design was used as this type of design is suitable to study issues in depth [15]. Qualitative design is fruitful in process evaluations as the qualitative inquiries can highlight matters broader than the anticipated outcomes, for example how the implementation was undertaken, informal patterns and unexpected interaction from a variety of perspectives [15]. Inspired by a previous study regarding weight management [16] the CFIR was used to guide data collection which was undertaken as focus groups and individual interviews, structure the data, and ultimately interpret the findings.

### Setting

The HSS intervention is conducted in school, but targets behaviours in the home environment. The programme is based on Social Cognitive Theory [17] and is carried out for 6 months in pre-school class where children are 5–7 years old. The curriculum in pre-school class is flexible and includes health topics. The HSS programme can therefore easily be integrated at this age. The programme consists of three core components;

1. Information to parents in the form of a brochure with easy-to-read advice and evidence-based information on diet and physical activity for children which was developed based on a literature review [18] and pre-tested by parents of children in the first grade in the targeted areas.
2. Motivational Interviewing (MI) [19] with parents, performed by skilled MI counsellors who were part of the research team, involving a 45-min individual session focusing on a specific aspect of the child’s dietary or physical activity behaviour, chosen by the parent. A follow-up session was offered three months later either face-to-face or by telephone.
3. Classroom activities for the children developed in collaboration with pre-school teachers. Ten 30-min lessons provided by the teaches according to a teacher’s manual, following the themes in the parental brochure, and accompanied by a workbook to complete at home together with their parents.

The programme was carried out in 13 schools with a total of 31 pre-school classes (intervention *n* = 16, control *n* = 15) during 2012–2013 as a cluster-randomised wait-list controlled trial in disadvantaged areas in Stockholm, Sweden, where the obesity prevalence is ten times higher than in areas with high SES (5 % versus 0.5 %) [20]. First, schools in the targeted areas were invited to participate. Second, all parents with a child beginning pre-school class in a school that had agreed to participate were invited. Parents were recruited via a letter, group meetings and in person by research assistants present at the schools in the mornings. In total 378 parents consented. The study design has been published [11]. The intervention was effective in decreasing the intake of unhealthy foods and drinks and BMI in obese children [21]. In the first MI session 146 parents participated, of whom 86 also participated in the second session. Teachers spent an average of 33 min on the classroom lessons and 8–10 lessons were performed by each class. Twelve of the 16 intervention classes completed all 10 home assignments whereas the rest completed between 1 and 8 of the assignments [21].

### Participants

A purposeful sample with maximum variation was chosen from the parents (185 families) in the intervention group of the HSS to capture a range of important characteristics [15], increasing transferability of the findings. Selected parental characteristics were: sex of the parent and child, country of birth, participating schools, family’s degree of participation, and target behaviour in the MI sessions, described in Table 1. Based on a variation of above characteristics, 45 parents were contacted first by mail and then by telephone and invited to participate. The focus groups were scheduled based on days and times suitable for the parents. About 75 % of the parents declined participation due to lack of time. All parents who had participated in at least one MI session (104) were therefore contacted by telephone and invited to participate in the study. For each of the four focus groups, 8–11 parents were recruited of whom 4–7 cancelled appointments without advanced notice or did not attend sessions they had agreed to participate in. In total 14 parents participated. Seven of the 13 intervention schools were represented in the sample. Of the 14 participants, one was a single parent and one was the parent of twins.

**Table 1:** Characteristics of parents

Parent	Sex of parent	Sex of child	Age	Education	No. of children	Country of birth	Years in Sweden	Number of MI sessions	Behaviour focus in MI session	School class
1	F	B	40	University	4	Sweden	-	2	Activity/food	A
2	F	G	35	High school	2	Sweden	-	2	Overweight/food	A
3	M	B	49	University	3	Iraq	23	2	Activity/eating together	B
4	M	B	43	Elem. school	4	Iraq	14	2	Sleep	B
5	F	G	41	University	2	Korea	40	2	Activity	C
6	F	G	34	University	2	Sweden	-	2	Parental influence on child	D
7	F	G	43	High school	2	Sweden	-	2	Activity/food	D
8	F	B + G	43	Elem. school	6	Lebanon	26	2	Vegetables	E
9	M	G	31	High school	3	India	15	1	Activity	F
10	F	B	35	High school	2	Turkey	15	1	Variation of food	G
11	F	G	34	High school	3	Sweden	-	1	Activity/food	H
12	M	B	39	High school	5	Afghanistan	10	1	No focus	F
13	M	G	37	University	2	Sweden	-	1	Activity	C
14	F	G	30	University	3	Somalia	18	1	Food	I

*F* female, *M* male, *B* boy, *G* girl, - = born in Sweden, Number of MI sessions corresponds to degree of participation in the intervention

Two indicators of low SES have been used in this study; area of residence, indicating SES on a group level and parent education, an indicator of SES at individual level [22, 23]. All three areas included in this study have a low employment rate and a low educational level [24], indicating low SES on a group level. These areas consist mainly of blocks of flats, a high proportion of inhabitants with a non-Swedish background and are also targeted specifically by the government to support socio-economic development [24]. In this study, parent self-reported educational level below university was regarded as low SES, described in Table 1.

All 21 teachers in the intervention group were invited to participate first by e-mail and then by telephone. Focus groups were scheduled based on days and times suiting the teachers. Ten teachers agreed to participate of whom all were women aged 29–53 years; four were primary school teachers and six were pre-school teachers who had worked in pre-school class from 2 to 25 years.

### Data collection

Data were collected through focus group methodology complemented with semi-structured interviews. Focus groups are a useful method in studies aiming to describe people’s experiences and data are generated through interaction between the participants in the group [25, 26].

Two interview guides were constructed, one for parents and one for teachers, according to the format suggested by Krueger [25]. Probing was used when appropriate and material from the intervention was used to facilitate discussion among the groups. The interview guides were pilot-tested on one parent and one teacher and consisted of open-ended questions based on the CFIR [13]. Examples of questions to teachers were: How did you join the programme? How did you perceive the work with programme themes? Did you adapt the material, and if so, how? How was the programme received at your school? In what way did the school leaders participate in the programme? What is your impression of the communication about the programme?

Examples of questions to parents were: How did you join the programme? How did you perceive the brochure? How did you perceive the MI session/s? What adaptations of the session/s are needed to suit you and your family? How did you perceive the work with the workbook? How did you perceive the dedication to the programme on the part of the school?

Parents and teachers were placed in separate groups to include participants with a common characteristic in the focus group [25]. The common characteristic of teachers was their role as deliverers of the intervention. For the parents, the common characteristic was the number of MI sessions they had attended. Parents who had participated in one MI session constituted one group, those with two sessions were another.

Four focus groups were conducted with parents, two with parents who had participated in one MI session (four parents in each group) and two with parents who had participated in two MI sessions (with four and two parents, respectively). Due to teachers’ time constraints only two focus groups were conducted with them (four in each group). In addition, two individual interviews were conducted where the same interview guide was used. The focus groups were conducted by a moderator (ÅN) and an assistant. Sandwiches and drinks were served during the focus groups and all participants were offered two cinema tickets for their participation. The participants filled out a short survey regarding age, education, country of birth, years of residence in Sweden, marital status and number of children in the family. All data were collected in October-December 2013 in four different schools. Data were audio recorded and transcribed by ÅN according to a set structure that also captured intonation, pauses and interrupted speech.

### Ethical considerations

Informed consent was collected from all participating teachers and parents. Ethical approval was obtained from the Regional Ethical Review Board in Stockholm, Sweden (2012/877-31/5) on the 14th of June, 2012.

### Data analysis

By listening to and transcribing the audio recordings and then reading transcriptions several times ÅN gained a thorough acquaintance with the data. Qualitative content analysis [27] was then performed in two steps according to Elo & Kyngäs [28]. First, relevant data corresponding to barriers and facilitators of the intervention were deductively sorted into two domains of CFIR: ‘Intervention characteristics’ and ‘Process’ [13]. Second, data sorted in each of the two domains were analysed inductively and each domain was analysed separately. Data indicating barriers and facilitators were identified and coded using the open coding technique suggested by Elo & Kyngäs [28]. Patterns among codes were found and codes were merged into subcategories. Next, patterns were found within subcategories which were merged into categories. Subthemes covering the inductive categories and an overarching theme covering all data were then identified. Data from teachers and parents were initially analysed separately. At the stage when subthemes were formed, inductive categories representing parents and teachers were merged and included under the three subthemes that were identified. Notes were taken throughout the analysis process, quotes were noted and a table of findings was kept and updated continuously. In the quotes, ellipses, modifications and explanations are presented within square brackets to increase comprehensibility. Intonation is signalled in italics. To ensure anonymity, participants in each focus group were assigned a number (1–4). When quoted in the text, each participant is labelled teacher, mother or father together with the assigned number. The focus group from which the quote was drawn is stated at the end of the quote. All focus groups and interviews were conducted and transcribed in Swedish. Translation into English was performed at the stage of forming inductive categories in the analysis. The analysis was conducted by ÅN and peer-reviewed by AB to ensure trustworthiness. All authors agreed on the categories and discussed subthemes and themes until consensus was reached.

## Results

An overview of the findings, including themes, subthemes, deductive and inductive categories is presented in Table 2. The overarching theme will first be described followed by the inductive categories which are presented together with their corresponding barriers and facilitators.

**Table 2:**
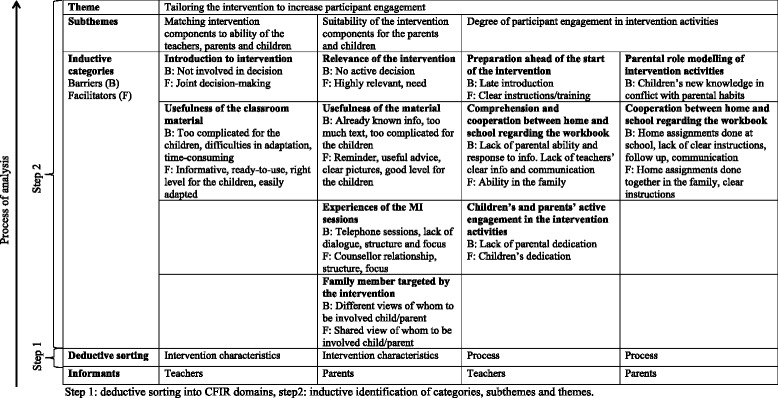
Description of analysis and findings

### Overarching theme–tailoring the intervention to increase participant engagement

The data revealed that the tailoring of the intervention to the abilities of the participants in terms of set up, degree of difficulty and presentation, affected participants’ engagement in the intervention activities.

The perception of the intervention as tailored to participant abilities or not, varied among the participants and influenced the degree of engagement among them. Parents who found that the intervention was not adequately tailored to their abilities, perceiving the components as either too difficult or too easy, seemed to lose enthusiasm over time. On the one hand, parents who found the intervention too difficult struggled to complete home assignments with their child. On the other hand, parents who found the intervention too easy simply took no notice of the health information as they felt they already knew it. Children who experienced that the intervention activities were tailored to their abilities showed interest and enthusiasm when engaging in the activities, which influenced the teachers positively.

When teachers perceived that the intervention was not adequately tailored to the parents’ abilities in terms of information and activities that were too difficult for the parents, they also experienced that the parents did not engage. This in turn decreased the teachers’ own engagement. Similarly, when the parents felt that the teachers did not respond to or use the workbook, their own engagement was affected negatively. Conversely, a positive attitude towards the intervention and high engagement was noted by both parents and teachers when the intervention components were perceived as being well tailored to the participants’ abilities, the teachers tailored the classroom lessons to fit their work situation, and the work book and home assignments were carried out as intended by children.

### Intervention characteristics-barriers and/or facilitators among teachers

#### Introduction to intervention

The way the teachers were introduced to the intervention seemed to affect how they engaged in it. Some of the teachers described being asked by their superiors, which seemed to facilitate implementation, whereas most portrayed that they were told to carry out the intervention. Few of the latter felt that they could refuse, which affected them negatively as they felt forced to conduct the programme without being allowed to reflect on whether they wanted to or not. Teachers explained:

Teacher 1: [if we] really are to do this [intervention once more] then we’re going to… *do it* […] so that it doesn’t become a *must* […] Because now it feels a bit like a *must* […]

Teacher 2: [The principal] came in spring and spoke about this ‘fantastic’ [imitating the principal’s excited voice] project and so on. And said we were going to work with it and …, there’s no saying *no* really. (Focus group 2).

#### Usefulness of the classroom material

Teachers’ perceptions of the design and the adaptability of the lessons were related to the children’s ability to perform the activities, which in turn affected the way the teachers conducted the programme.

Teachers who found the lessons to be on approximately the right level for the children also perceived that the lessons functioned well, were fun and easy to adapt to the children’s levels. They perceived the teachers’ manual and the material as informative and ready-to-use in class, with clear instructions and good structure. They performed the activities successfully and viewed the manual as a starting point and then planned the lessons according to the needs and preconditions of the children or time constraints. Teachers discussed:

Teacher 3: It was easy; you got a free lesson [laughing]. You could simply talk about these sentences [in the teachers’ manual].

Teacher 2: Yeah, these issues you were supposed to raise were easy to take up with the children, they got engaged by the questions. It was right on the children’s level, a perfect fit for six-year-olds.

Teacher 1: Yeah, it’s about their everyday life in a way and so it’s close at hand for them. […] And then it lay a good foundation, I know that we did a lot in relation to this as well. If we got an idea from the manual [… then we could] use a lot of other material, bring it in to make it clear for the children. (Focus group 1).

Other teachers perceived the children’s abilities to perform the activities as inadequate in relation to how the lessons were formed. They perceived the lessons as too time-consuming if performed according to the manual and felt a need to simplify a lot as they found the lessons too complicated and at times irrelevant, making the intervention unsuitable for their classes. A teacher explained:

But… they were *little* kids*…* So if you… If you take too *much* of like the actual… manual here where there are explanations and that […] it’s too *difficult* for six-year-olds… too much information, so you have to *remove* so much [of the lessons’ contents] I feel. (Interview 1).

### Intervention characteristics–barriers and facilitators among parents

#### Relevance of the intervention

Perceiving that the intervention focused on their families’ everyday life, and deciding to participate due to a need, both seemed to facilitate active engagement in the intervention. Parents who decided to participate just ‘because everyone else did’ seemed less engaged.

#### Usefulness of the material

The contents, design and complexity of the material all appeared important in relation to the different levels of ability among parents and children. Several parents viewed the material, especially the brochure, as too basic, which made them disregard the information, whereas others thought that the information served as a good reminder. Some also described the brochure and workbook together as good summaries, containing useful advice, easily accessible and clear. Parents discussed:

Mother 1: This information that you get. Sure it’s good to get but it’s really a thing that yeah… goes in one ear and out the other.

Father 1: That book [brochure] actually helped you think about like, ‘ooh, this is good for children’, so that even if you knew what they should eat, it was an extra help because you remember what to tell them. (Focus group 7).

Some reflected on other parents’ abilities, calling for translation into more languages, less text and more pictures in order for the material to be accessible to parents who do not speak very much Swedish.

The children’s abilities in relation to the material was also discussed; some parents perceived the workbook as too complicated for six-year-olds, whereas others found it a good fit for the children but called for repetition in first grade.

#### Experiences of the MI sessions

The parents experienced the MI sessions differently and varied in their descriptions of a good relationship with the counsellor and structure of the session.

Some parents described being listened to and appreciated the specific focus on their own needs and worries. They perceived the session structure as clear and felt it was useful choosing a specific behaviour change and sometimes setting a goal during the first session followed by a second session to assess the goal. Parents discussed:

Mother 1: Then [during the MI session] we could talk about him, it wasn’t general, it was his own particular problems. And then I think you are influenced more by a conversation, because it’s a relationship then and that influences more.

Mother 2: Well, it was up to the parent to adapt the conversation, I felt at least. I got to adapt the conversation according to the needs I had for my child. (Focus group 5).

Other parents seemed to have had expectations on MI that were not met by the structure of the session and the counsellor’s behaviour. These parents seemed confused by the session, which left them dissatisfied and frustrated. A mother explained:

Mother 2: I was expecting that *she* would tell me how we can *do* what we can *do*, but it was the other way *around*, it was me telling her how *I* do things, what I *do*, and so on. (Focus group 7).

Telephone sessions influenced the relationship with the counsellor negatively, as the parents felt it was difficult to take time to think, pause and express himself or herself thoroughly without the counsellor present in person.

#### Family member targeted by intervention

The HSS intervention mainly targeted parents, not children, and in many families only one parent participated actively. This seemed to affect the parent’s perception of whether the intervention was a good fit for their families.

Participants in families where the parents shared household responsibilities such as cooking, found it important for both parents to participate, to enhance discussion and cooperation between the parents whereas those parents who had the sole responsibility for household chores did not. Parents discussed:

Mother 1: If only one parent [participates], it has to be both [participating]. At least if both cook. That both participate in the project and […] that it [the intervention] encourages parents to discuss with each other because you sort of have to cooperate.

Mother 2: Personally I didn’t feel any need for my husband to come along [to the MI session] because we discussed everything that was brought up in that conversation and what was going on in the project. (Focus group 5).

Parents expressed different views regarding the children’s involvement in the intervention components. Some wished for their child to be the main participant in the programme, especially in the MI sessions. These parents thought their child was the one who needed the information about diet and physical activity, not themselves as parents. Others did not want their child present, as they wanted the issues to be natural to the child and thought of it as a parental responsibility. Parents discussed:

Father 1: Because at *some* level I think children have to experience it in a *natural* sort of way, that food shouldn’t be this big *issue* […] And for *that* reason I *don’t* think it’s good that the children participate [in the MI session].

Mother 1: Well I have a different opinion, of course children can participate. And well, for me it’s natural since, in the end, it’s the children who are going to learn and be there and listen.” (Focus group 8).

### Process-barriers and facilitators among teachers

#### Preparation ahead of the start of the intervention

Teachers’ possibilities to prepare for the intervention affected their engagement in it.

Clear instructions about how to carry out the lessons were perceived as positive. However, the intervention was first presented when the teachers had already finished their planning for the school year, and would have been more integrated in the school work if it had been presented earlier.

#### Comprehension and cooperation between the home and the school regarding the workbook

Proper comprehension of the home assignments on the part of the families as well as good cooperation between home and school regarding the intervention had a substantial influence on participant engagement.

The teachers experienced a lack of parent-teacher communication about the programme in general and about the home assignments in particular.

The teachers identified the parents’ ability to understand the assignments, support the children and manage to encourage the child to bring the workbook to and from school as being important for success. Teachers who perceived parental ability as adequate also noticed how the families completed the assignments together. Other teachers identified low parental ability to understand and support their children with the home assignments, at times to the extent that the teacher chose to complete home assignments at school instead of sending them home. The low parental ability was demonstrated by children who had completed the assignment improperly or clearly not understood it. A teacher explained:

One child came back and had done *everything* [in the workbook]. The first time [lesson]. Then you wondered ‘but what were you thinking there, Mommy or Daddy’. Then, then you don’t understand a thing. And yet we were… I was so-o-o-o clear. I know I *have* to be. To [explain] this is lesson number one, and it’s *this* assignment we have now. (Interview 2).

#### Children’s and parents’ active engagement in intervention activities

Teachers described different child and parental engagement in the activities connected to the classroom lessons and home assignments.

Most teachers faced interest and positive dedication on the part of the children but lacked interest and response from the majority of parents. The children’s response stimulated the teachers in their work whereas the parental lack of response resulted in disappointment. Teachers discussed:

Teacher 2: No, I think we invested *a lot of* time and I thought I prepared a lot and it was fun with the children, but then got *no* response from the parents. Is what I felt. Unfortunately……

Teacher 1: Yes, but I think they [the children] thought it was fun.

Teacher 3: Yes, me too as I remember it. And they participated when we discussed these issues, and *knew* lots of things. (Focus group 2).

### Process-barriers and facilitators among parents

#### Parental role modelling of intervention activities

Parents’ failure to act as good role models in relation to the home assignments and the children’s new knowledge regarding health behaviour affected implementation negatively. Parents described how things the children learned in the programme clashed with their own priorities. For example, lack of time or stress resulted in parents driving the child places, despite the children’s wish to walk. The children’s interest in the Keyhole^1^ was experienced as disturbing the parents’ own food habits. Some parents showed that the Keyhole was not important, either by convincing the children or showing a lack of interest. A mother described:

Mother 2: I had [the child] with me in the grocery once. I wasn’t allowed to buy anything that didn’t have the Keyhole. That didn’t go very *well* if you’re *used* to buying *other* stuff that maybe there absolutely isn’t even *any* kind with the Keyhole. ‘But Mom we can’t buy that, we have to have the Keyhole’ [imitating the child]. ‘Yes but it’s fine, *today* it’s okay [to buy (imitating her own persuasive voice)]. (Focus group 6).

#### Cooperation between home and school regarding the workbook

Cooperation between home and school, especially regarding the workbook, proved important for parental engagement in the activities.

In general, the parents experienced little communication about the programme with the school. The degree to which the workbook was sent to and from school varied, and this influenced the parental and family engagement. Many parents were disappointed that the workbook was completed in school and not sent home as intended, as this prevented them from engaging in the intervention. Other parents lacked structure regarding the assignments or lacked follow-up on the part of the school, which resulted in poor adherence to the workbook activities. Some parents expressed that the teachers provided clear and helpful instructions and that working with the assignments at home facilitated change in family habits. Parents discussed:

Mother 1: They [the home assignments] activated the entire family to activity I’d say. Lots of fun.

Father 1: I guess the… form teacher was supposed to be in charge of these and hand them out as home assignments in some way […] it felt like it was *allowed to slide* a bit after a while to be honest […] So *this* [workbook] feels like something that was *diluted* after a while.

Mother 1: We got it as home assignments. She wanted to do her assignment and draw and then, I think, the teacher made some mark, put on a star.

Father 1: But *that* was good.

Mother 1: …that, that I think, that was good.

Father 1: They should have done that in our class too I think. (Focus group 8).

## Discussion

This study investigated barriers and facilitators of a parental support programme as perceived by teachers and parents in disadvantaged areas. The findings reveal that the degree of tailoring of design and degree of difficulty regarding the intervention components influenced the level of participants’ engagement in the intervention activities.

The findings indicate several aspects in need of attention when developing parental support programmes for disadvantaged areas where school and parents interact. These aspects include the need to tailor components to the abilities of parents with low SES, to enhance parenting skills and cooperation, and improve the interaction between the home and school settings.

In the process evaluation of the first HSS trial conducted in less disadvantaged areas we found that there was a need for better communication and clearer roles between parents and teachers [14]. In the current study, cooperation between home and school was also highlighted. In addition, the need for further tailoring of the intervention in order to reach higher engagement among the participants was emphasised. This indicates that lack of adequate tailoring of the HSS programme is a barrier in settings with low SES.

### Tailoring a programme that ‘fits all’ in disadvantaged areas

In the CFIR, Damschroeder et al. [13] identify how both the complexity and the adaptability of an intervention can hinder or facilitate implementation. Interventions that are tailored to the target group are more easily implemented, whereas poorly tailored or overly difficult intervention components may provoke resistance from the participants [13]. Our findings indicate the importance of tailoring intervention components to match participants’ abilities.

#### Tailoring written material

The teachers’ manual with clear instructions and ready-to-use activities and material was well tailored to the abilities and interests of the children according to several teachers. This is in line with results from previous studies in implementation of health promotion interventions in schools, showing that components which are easy for teachers to use, do not require much time to prepare and that fit the rest of their school work, have been found successful [7–9, 29].

A number of teachers, however, found the material too complicated for both children and parents, which was also raised by some parents. Teachers varied in their descriptions of both parents’ and children’s abilities to carry out the programme and in their perceptions of whether the programme was suitable or too complicated for their classes. Some also seemed to find it difficult to tailor the work to the children’s ability levels, which resulted in them not sending home the workbook, an omission that reduced fidelity to the intervention.

This may indicate a lack of tailoring of the material to different abilities in the targeted families which has been seen in other school-based programmes targeting diet and physical activity [8]. This issue could be addressed by including clear distinctions between alternatives with different degrees of difficulty in the material or by clarifying core components and intervention content open for adaptation to both deliverers and participants.

#### Tailoring individual counselling

Studies using MI with parents in general demonstrate that parents appreciate the method and feel supported in behaviour change [30–32]. In a parental support programme targeting overweight and obese children in the US, parents with lower income and/or born outside the US were more satisfied with MI than other parents [33]. MI is a goal steering, client centred style of conversation designed to facilitate behaviour change [19]. In the HSS, MI was used in individual counselling with a focus on the needs of the parents, and thus tailored to the varying abilities and levels of knowledge that parents may have. In this study we saw that several parents perceived the MI sessions as useful, facilitated by a good relationship with the counsellor, structure and focus on the parent’s own perceived need. However, some felt confused about the structure of the sessions despite being well informed through telephone calls about the way the sessions would be carried out. These parents had unfulfilled expectations that the MI counsellors would do most of the talking during the session and offer advice. The HSS was conducted in areas where parents were born in a wide range of countries. Previous studies on cultural expectations on interaction with health care providers have revealed that ethnic origin may have an impact on successful health interaction [34]. Patients within some ethnic groups may cherish their individual autonomy, whereas other groups favour respect and hierarchy. The latter may expect health care providers to be firmer in giving advice and instructing the patient whereas the former may expect opportunities to reflect and make their own decisions [35, 36]. We believe that when one is using MI in a disadvantaged setting with ethnic diversity, MI techniques may need further adjustment to meet these specific expectations from the patients. When counselling a person with greater expectation of receiving advice, emphasis could centre on the MI technique ‘exchanging information’ (Elicit-Provide-Elicit) [19], where the counsellor asks permission to offer advice relevant to the parent’s specific situation.

### Emphasising parental focus

The findings of our study indicate that parents’ perceptions of their own role and responsibility are important to successful implementation. When evaluating participation in parenting programmes, Mytton et al. [10] found that some parents refused to acknowledge the importance of parental involvement in children’s behaviour changes. This is similar to our findings where some parents wished for the children to participate in the MI sessions and failed to identify the importance of themselves as role models. These parents expressed how the children were the ones who needed knowledge not themselves as parents. This may indicate that even though the main focus of the intervention is to influence diet and physical activity, additional emphasis on general parenting skills may be fruitful in these types of parental support interventions, for example regarding parents as role models.

Another challenge for parental support programmes is the inclusion of both parents, which was voiced by several parents in this study. A previous study of the MI session in HSS revealed that a major barrier to behaviour change regarding diet and physical activity for the children was negative interplay between parents: lack of cooperation or contradicting each other [37]. It thus seems necessary to include both parents to facilitate cooperation in parental support programmes.

### Combining the school and home setting in interventions

The findings indicate that successful implementation of a school-based parental support programme largely relies on good cooperation between home and school. Teachers and parents described how the programme was split between the school and home without much communication, and both parties called for greater dedication from the other. This was also found in the process evaluation of the previous HSS intervention conducted in a less disadvantaged area [14]. Efforts must thus be made to establish good cooperation and communication between the school and home in order to succeed with such a programme. We suggest a greater focus on preparing the teachers and parents for participation. Adding a ‘start-up’ component with structured activities to create cooperation among parents and teachers, enhancing insight in each other’s roles and needs, may be something to consider.

## Strengths and limitations

This study is one of the first evaluating the process of an intervention actively involving both the school and the home setting in a disadvantaged area. The structured use of the CFIR in this study enhances transparency of the findings and could possibly increase transferability to similar contexts.

It was difficult to recruit parents to the study as many declined due to time restraints, cancelled appointments without advanced notice or did not attend sessions they had agreed to participate in. Recruiting participants in settings with low SES has proven difficult in previous studies [38, 39], which is problematic when it comes to representativeness of the target group. The participating parents in this study may represent the most interested parents in the HSS intervention; relatively well educated and none had resided in Sweden less than 10 years, which may indicate sampling bias and lack of representativeness of the parental group in the HSS study. However, despite these difficulties we believe it is worth conducting this kind of study in low SES settings, as if future research is not undertaken, little will be known in regard to perceptions and needs in disadvantaged groups. The participants of this study were offered two cinema tickets and it is possible that other incentives could have been more successful. In addition, using individual interviews in a place chosen by the parent or by telephone could facilitate recruitment to this type of study.

## Conclusion

The study points to the importance of tailoring intervention components to the abilities of the target group in order to increase participant engagement in an intervention.

Including activities that focus on parents as role models and cooperation between parents also seem important to bring about changes in the home environment as well as cooperation between home and school when the programme is based in the school setting. The findings of this study may contribute to the developments of effective parenting programmes among groups with low SES. The findings of this study inform further development of school based parental support programmes in general and in low SES groups specifically. The findings could also contribute to a higher success rate in obesity prevention and have a positive impact on equality in health.

## Abbreviations

CFIR: Consolidated Framework for Implementation Research
HSS: A Healthy School Start
MI: Motivational Interviewing
SES: socio-economic status

## Footnotes

1 The Keyhole is a nutrition label developed by authorities in Norway, Sweden and Denmark to indicate grocery store products that contain less fat, salt and sugar and more whole grain and fibre than the average for that particular foodstuff.
